# Identifying unique spectral fingerprints in cough sounds for diagnosing respiratory ailments

**DOI:** 10.1038/s41598-023-50371-2

**Published:** 2024-01-05

**Authors:** Syrine Ghrabli, Mohamed Elgendi, Carlo Menon

**Affiliations:** 1 Biomedical and Mobile Health Technology Lab, ETH Zurich, 8008 Zurich, Switzerland; 2 Department of Physics, ETH Zurich, 8093 Zurich, Switzerland

**Keywords:** Diagnostic markers, Diagnosis, Public health, Biomedical engineering, Electrical and electronic engineering

## Abstract

Coughing, a prevalent symptom of many illnesses, including COVID-19, has led researchers to explore the potential of cough sound signals for cost-effective disease diagnosis. Traditional diagnostic methods, which can be expensive and require specialized personnel, contrast with the more accessible smartphone analysis of coughs. Typically, coughs are classified as wet or dry based on their phase duration. However, the utilization of acoustic analysis for diagnostic purposes is not widespread. Our study examined cough sounds from 1183 COVID-19-positive patients and compared them with 341 non-COVID-19 cough samples, as well as analyzing distinctions between pneumonia and asthma-related coughs. After rigorous optimization across frequency ranges, specific frequency bands were found to correlate with each respiratory ailment. Statistical separability tests validated these findings, and machine learning algorithms, including linear discriminant analysis and k-nearest neighbors classifiers, were employed to confirm the presence of distinct frequency bands in the cough signal power spectrum associated with particular diseases. The identification of these acoustic signatures in cough sounds holds the potential to transform the classification and diagnosis of respiratory diseases, offering an affordable and widely accessible healthcare tool.

## Introduction

Coughing, a multifaceted symptom observed in a range of illnesses from cystic fibrosis and pneumonia to chronic obstructive pulmonary disease (COPD) and bronchitis, presents unique diagnostic challenges due to its variable severity and manifestation^1,2^. Traditional diagnostic tools for respiratory diseases, such as chest scans, swab tests, and bronchoscopies, are effective but often lack accessibility and affordability^3–6^. This gap underscores the need for alternative solutions, where artificial intelligence (AI), particularly machine learning, emerges as a promising avenue, offering more accessible and cost-efficient diagnostic methods using devices as common as smartphones^7–10^.

Intriguingly, a study by Smith et al. revealed that even healthcare professionals struggle to diagnose underlying conditions based solely on cough sounds, with accuracy rates as low as 34% for clinical diagnoses^11^. This challenge is further complicated by the similarity in cough sounds across different ailments to the human ear, despite the presence of distinct acoustic variations detectable by machine learning models^12–14^. However, the utilization of these models in healthcare is hindered by their lack of interpretability and explainability^15^.

Our research delves into the physics and acoustics of cough sound signals, aiming to map these signals to their corresponding respiratory diseases and explore coughing as a potential biomarker. We focus on identifying specific spectral features within cough sounds for respiratory disease diagnosis, extending our study to differentiate cough signals in pneumonia and asthma patients from those in healthy individuals^16,17^. This approach not only aims to enhance the understanding and application of AI in healthcare but also to fill a significant research gap in cough biomarker identification and the acoustical properties of cough for disease diagnosis^17–24^.

## Methods

This study focused on uncovering distinct spectral features in cough sound signals to aid in the diagnosis of respiratory diseases. Our methodology involved two primary steps: conducting separability tests and applying machine learning classification techniques to analyze the relative power (RP) of these cough sound signals.

Initially, we embarked on a preliminary assessment of the cough signals. This involved calculating the power density distribution of the coughs. Our initial findings indicated noticeable differences in the frequency of cough power between patients diagnosed with COVID-19 and those in a control group.

Building on this preliminary data, we delved deeper into the spectral power distribution characteristics of cough signals stemming from various respiratory conditions. To accomplish this, we employed sophisticated machine learning algorithms, specifically linear discriminant analysis (LDA) and K-nearest neighbors (KNN). The aim here was to pinpoint specific frequency bands in the power density spectrum that are uniquely associated with different types of respiratory ailments.

### Data and data processing

During the COVID-19 pandemic, multiple organizations gathered data on the coughs of COVID-19-positive patients via internet platforms, smartphone apps, or web apps^9,10,25^. This produced copious amounts of crowdsourced cough audio data, which are now publicly available and documented^26–28^. For this study, we used the Coswara dataset^29^, which contains slightly more than 1000 samples of respiratory sounds gathered between April 13, 2020, and February 21, 2022 via users’ computer microphones (crowdsourcing). The dataset is comprised of 61$\%$ male patients, and its majority (90$\%$) is from Indian ethnicity, spans 15–90 years old of age. Notably, approximately 45% of the participants fall within the age range of 15–30 years old, while 28% are between 30 and 45 years old. This age distribution reflects a diverse sample, allowing for the examination of cough sound signals across different age groups. Additionally, Coswara compiled metadata from user survey data on its web platform^30^, which enabled us exclude healthy and non-symptomatic patients and to establish a COVID-19-negative control group comprising ill patients who had cough as a symptom of their maladies. The patients’ coughs used for this study were thus all affected by any of the various respiratory illnesses, such as the common cold, COPD, viral rhinitis, pneumonia, and asthma.

After selecting the relevant data from the dataset, we applied an audio segmentation algorithm to all signals. This was to separate individual cough events from a single audio file that might contain several coughs. The segmentation was achieved through a combination of time-domain and machine learning-based methods. Time-domain segmentation involved breaking down the audio signal based on characteristics like amplitude in the time domain, facilitating the isolation of relevant audio segments. Machine learning-based segmentation utilized pre-trained convolutional neural networks (CNN) to identify cough events. The segmentation process was validated through iterative parameter tuning, selecting the most effective algorithmic alteration. This step ensured that noise, extraneous breath, voiced sounds, and any background audio were minimally present in the spectral analysis.

Based on the recommended cut-off frequencies found in the literature^19,20,31,32^, we chose to use a low-pass filter with a cut-off frequency of 4000 Hz. This step is a crucial part of the preprocessing in every pipeline we apply.

### Spectral analysis

Power spectral density (PSD) is given as a frequency-domain plot^33^ showing the strength of the fluctuation (energy) as a function of frequency. In a time series, given an audio signal, *x*(*t*), and its Fourier transform *f*(*q*), the average power of the signal and the PSD are respectively defined as^34^: $P= \lim _{T \rightarrow \infty } { \frac{1}{T} \int _{-\infty }^{\infty } |{f}_{T}(q)|^2\,dq }$ and $S= \lim _{T\rightarrow \infty }\frac{1}{T}|{f}_{T}(q)|^2$.

The Python Scipy library^35^ provides computation for Welch and periodogram methods^36,37^, which enabled us to estimate the PSD, using its default parameters, such as Hann window, on the audio data that was sampled at a frequency of 22.5 kHz and of approximately 10 ms of duration each. We then used the PSD values to compare the COVID-19-positive and COVID-19-negative coughs. For this, we generated a PSD plot using cough sound signals not only to compare COVID-19-positive and COVID-19-negative coughs but also to isolate asthma and pneumonia subgroups of COVID-19. We then considered the PSD plot to compare asthma and pneumonia with COVID-19.

### Separability test

For a certain frequency band $(F_1,F_2)$, we calculated the relative power of cough sound signals for the groups as follows:

- Each audio file was passed through a band-pass Butterworth filter (with roll-off at 6dB/octave) to retrieve the signal in a certain frequency band $(F_1,F_2)$.
- The relative power (RP) of the signal extracted at each interval of frequency was derived from the formula $RP_{i, 1-2}= \frac{ P_{i}(F_1-F_2) }{ P_{i}(F_0,F_{f})}$ where $P_i$ the power value of a sample *i* of audio from the PSD and $F_0=1$ Hz and $F_f=4$ kHz. The wide band $[F_0\smallsmile F_f]$ defines a low-pass filter we performed on all sound signals to eliminate noise.
- The average RP $\mu$ was computed for all cough audio samples, each relevant category of illness, and the control group. The standard deviation was denoted $\sigma$ and similarly computed. The linear separability criterion was denoted *J* and equal to^38^:
  $$\begin{aligned} J(F_1,F_2)={\vert \mu _{Ctr}(F_1,F_2)-\mu _{Exp}(F_1,F_2)\vert \over \sigma _{Ctr}(F_1,F_2)+\sigma _{Exp}(F_1,F_2)}, \end{aligned}$$

where as previously mentioned, $\mu _{Exp}$ and $\mu _{Ctr}$ are defined as the average RP for the relevant experimental and control groups, respectively, and $\sigma _{Exp}$ and $\sigma _{Ctr}$ as the standard deviations within the signals of the experimental and control groups, respectively.

### Linear discriminant analysis classifier

Linear discriminant analysis (LDA) is a statistical method used for classification and dimensionality reduction. It is a supervised learning technique that seeks to find the linear combination of features that maximally separates different classes of data. LDA assumes that the data are normally distributed and that the classes are linearly separable, meaning that they can be separated by a single linear boundary. The algorithm initially calculates the means and variances of the features for each class, which are then used to determine the optimal linear boundary between classes. We applied the LDA algorithm to the RP values of the COVID-19-positive group versus the control group using several frequency bins to determine whether they were, in fact, linearly separable. We repeated the experiment for pneumonia versus asthma analysis.

### Nearest neighbors classifier

KNN is a supervised learning technique that classifies data by finding the k closest data points in a feature space to a given test point, and then classifying the test point based on the majority class among its k nearest neighbors. Unlike LDA, KNN assumes no underlying data probability distribution or linearity of the decision boundary between classes. Analogous to the LDA algorithm, we applied the KNN algorithm to the RP values of both the pneumonia and asthma groups (without confounding COVID-19), as well as to the COVID-19-positive and control groups, to determine whether they were indeed separable within the frequency bands of interest.

## Results

Initially, we studied the PSD values, focusing on analyzing paired samples of COVID-19 versus the control group, pneumonia against asthma, and COVID-19 against both asthma and pneumonia. To ensure clarity, Table 1 details the number of segmented cough audio samples used for the computations and the number of patients from the Coswara dataset reported to be affected by each of the relevant illnesses. Patient numbers and samples numbers are different in that the samples are constituted of the isolated audio of a cough event: a total of 1183 cough segments of COVID-19 positives versus 341 audio segments in the COVID-19 negative category. To palliate to imbalance in classes we used under-sampling.

**Table 1:** Number of patients in dataset categories composition.

	Ssthma	Pneumonia	COVID-19 sans asthma/pneumonia	Illnesses sans COVID-19	Total
COVID-19 positive	19	11	367	0	397
COVID-19 negative	26	18	0	127	171
Total	45	29	367	127	568

The patients and samples differed in that the samples consisted of isolated audio recordings of cough events. Each patient’s audio file contained several such events.

**Figure 1:**
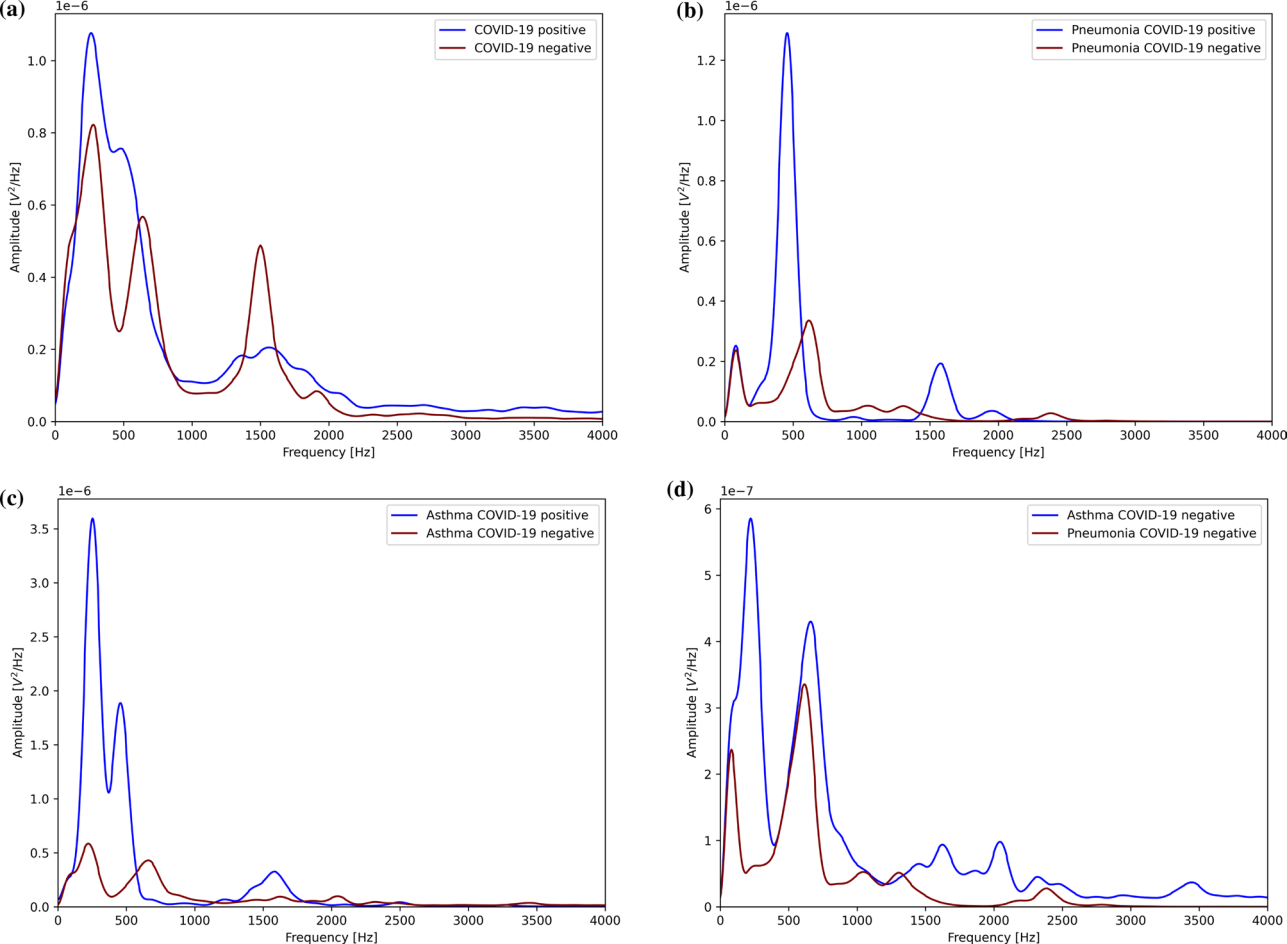
Power spectrum density (PSD) computed on each audio using the Welch method and averaged for all population cough signals extracted from the crowdsourced publicly available dataset Coswara^29^. The dataset’s recordings were collected from participants using their built-in computer microphone, the sampling rate of the data is 22,050 Hz. (**a**) Comparison of PSD between cough signals of COVID-19-positive and COVID-19-negative patients. Zoom-in view between 1 and 4 kHz. (**b**) Comparison of PSD between cough signals of pneumonia COVID-19-positive and COVID-19-negative patients. Zoom-in view between 1 and 4 kHz. (**c**) Comparison of PSD between cough signals of asthma COVID-19-positive and COVID-19-negative patients. Zoom-in view between 1 and 4 kHz. (**d**) Comparison of PSD between cough signals of patients with asthma and pneumonia. Zoom-in view between 1 and 4 kHz.

**Figure 2:**
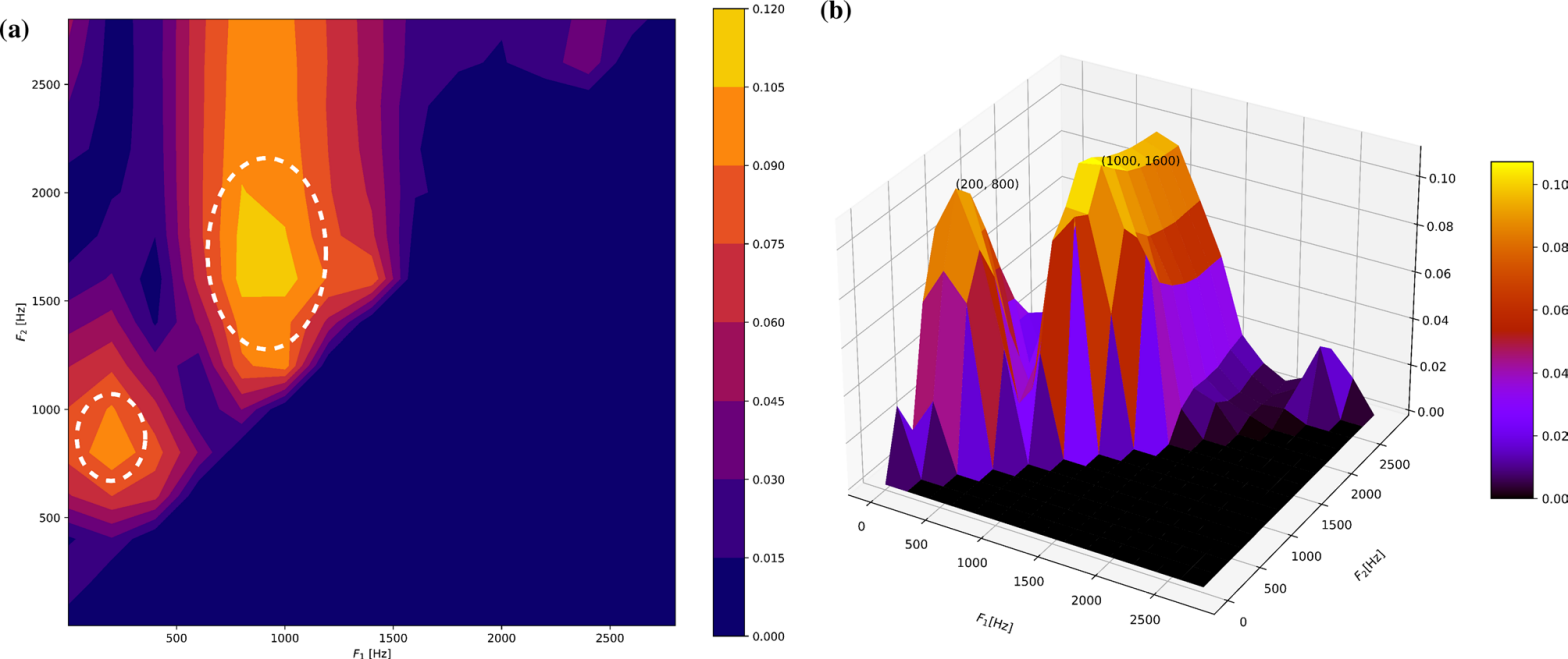
2-D and 3-D visual representations of the linear separation value *J* optimization process for distinguishing between COVID-19-positive and COVID-19-negative patients using cough signals, extracted from the publicly available Coswara dataset^29^. (**a**) 2-D visualization of the optimized separation value over different frequency bands, demonstrating the clear differentiation between COVID-19-positive and COVID-19-negative patients. The white dotted lines on the 2-D plot indicate the boundaries of distinct regions-Region 1 (left) and Region 2 (right). (**b**) 3-D visualization of the optimization process, providing a more comprehensive view of the separation between the two groups and highlighting the potential of utilizing cough signals as a diagnostic tool for COVID-19. The dataset’s recordings were collected from participants using their built-in computer microphone, the sampling rate of the data is 22050 Hz.

A preliminary exploration of the PSD values, displayed in Fig. 1, revealed observable differences between the curves for each pair of categories. Figure 1a shows a comparison of the PSD values for COVID-19-positive patients’ coughs and the control group’s coughs. The first noticeable difference occurred in the range of frequencies between 100 and 800 Hz, and the second in the frequencies approaching 1500 Hz. These frequencies are in the low- and high-frequency ranges, respectively. The COVID-19-positive PSD curve revealed a peak at 250 Hz, which was higher in amplitude than that revealed by the COVID-19-negative curve-a characteristic that we observed in the COVID-19-positive curves of all three plots (see Fig. 1a–c). A second peak at around 1500 Hz is also distinguishable in all three figures, which was half the amplitude of the peak of the control group, as seen in Fig. 1a. These features allowed us to separate COVID-19 from other illnesses, and vice versa, within the two regions of frequencies corresponding to the previously mentioned low- and high-frequency ranges. Comparing asthma and pneumonia without confounding COVID-19, we observed a difference between the two curves at the frequencies of 80–220 Hz (a relatively very low frequency range) and 616–660 Hz. We also observed similar differences in the three peaks of asthma-infected patients at 1616 Hz, 2040 Hz, and 3450 Hz. The final peak at 3450 Hz in the PSD curves of the asthma samples was the lowest and negligible for our analysis. The differences in the PSD curves were indicative of patterns in the cough sound signals (i.e., notable and distinguishable in COVID-19-positive patients) and potentially valuable for use with a diagnostic machine learning classifiers. Comparing COVID-19-positive and COVID-19-negative asthma and pneumonia samples provided secondary support for related frequency bands capable of isolating COVID-19 from other respiratory illnesses. It also highlighted that asthma and pneumonia curves exhibited differences in lower and higher frequencies, indicating distinct patterns in the cough sounds associated with these illnesses.

The linear separation value $J(F_{1}, F_{2})$, estimated in the range 1–3000 Hz in Fig. 2 consistently showed two distinct regions with large *J* values indicating higher separability. The first region we observed in Fig. 2a aligned with the PSD values highlighted previously, spanning the 1–1500 Hz frequency bands. The second was between 1000 and 2500 Hz. These identified regions will be labeled Region 1 and Region 2 for the rest of our analysis. The three-dimensional plot of the linear separation value $J(F_{1}, F_{2})$, calculated within the frequency range of 1–3000 Hz, is presented in Fig. 2b. The maximum *J* values is highlighted with a tick in the plot. In Fig. 2b, the maximum of the separability value is in the 1000–1600 Hz band, and the second maximum *J* value (i.e., the second peak) is located in the 200–800 Hz band. Both regions tally with the previous observations shown in Fig. 1. In summary, the representations of the *J* separability criterion of the cough sound signals’ RP values shown in the plots in Fig. 2 provide a comprehensive view of the patterns and frequency bands of interest, support our preliminary observations of the PSD curves in Fig. 1, and could be valuable for developing diagnostic algorithms.

**Table 2:** *p* values for the COVID-19-positive group versus the COVID-19-negative control group and the pneumonia group versus the asthma (COVID-19-negative) group.

Region 1		Region 2	
Frequency band in Hz	*p* values of COVID-19-positive vs. COVID-19 negative	Frequency band in Hz	*p* values of COVID-19-positive vs. COVID-19 negative
$\mathbf {[300, 400]}$	$\mathbf {6.53\times 10^{-4}}$	$\mathbf {[1100, 1650]}$	$\mathbf {4.58 \times 10^{-4}}$
[300, 600]	$1.99\times 10^{-3}$	[975, 1750]	$5.89 \times 10 ^{-4}$
[300, 800]	$9.35 \times 10^{-3}$	[1100, 1850]	$5.92\times 10^{-4}$

Region 1		Region 2	
Frequency band in Hz	*p*-values of asthma vs. pneumonia	Frequency band in Hz	*p*-values of asthma vs. pneumonia
$\mathbf {[450,1000]}$	$\mathbf {1.11 \times 10^{-2}}$	$\mathbf {[1400, 1600]}$	$\mathbf {1.39 \times 10^{-3}}$
[50, 900]	$4.54 \times 10^{-2}$	[1450, 2100]	$8.74\times 10^{-3}$

We computed *p*-values using a Mann–Whitney U test to analyze the RP of signals within frequency bands of interest.

The next step involved assessing the *p* values resulting from the Mann–Whitney U test^39^. The t-test can be less reliable with unequal sample sizes, however, Mann–Whitney U test does not have this limitation^40^. In addition, due to the non-parametric nature of Mann–Whitney U test, it makes the analysis more generalizable^41^. Table 2 presents the results of the statistical test’s heuristic search and consequently only includes the minimum values returned by the U-test. The frequency bands featured are encompassed by the same frequency regions as described in Fig. 1 (around 300–800 Hz and 1100–1850 Hz for the RP distributions of the COVID-19-positive versus COVID-19-negative control group, and around 100–800 Hz and 1400–2100 Hz for the frequency bands of the asthma and pneumonia categories without confounding COVID-19. The *p* values ranged from $6.53\times 10^{-4}$ to 0.0454 and were below .05 for all the frequency bins we computed (i.e., 300–400 Hz and 1100–1650 Hz for the COVID-19-positive group versus the COVID-19-negative group, with *p* values equal to $6.53\times 10^{-4}$ and $4.58\times 10^{-4}$, respectively. The first optimal frequency band was within Region 1, and the second was within Region 2. The optimal frequency band was 1400–1600 Hz for the computation of the asthma versus pneumonia *p* values, with a value of $1.39\times 10^{-3}$. For comparison, the COVID-19-positive versus COVID-19-negative and the asthma versus pneumonia *p* values for the RP in the frequency band of 1–3000 Hz were 0.49 and 0.66, respectively.

Both the low and high *p* values presented herein are consistent with our preliminary observations and showed significant differences in the RP values between the COVID-19-positive and COVID-19-negative control groups. They also supported the existence of frequency bands capable of separating each of the categories, in line with our preliminary observations and demonstrating the presence of an acoustical signature for the medical conditions causing coughs. We devoted the final part of the study to classifying the RP values of the optimal frequency bands, as discussed previously, using LDA and KNN machine learning classifiers. It is crucial to highlight that the availability of samples for COVID-19-positive and COVID-19-negative cases significantly influenced our data selection strategy. To ensure the integrity and impartiality of our analysis, we deliberately opted for the random selection of a representative subset equal to 341 samples for each class, thereby mitigating potential biases in our findings. The pneumonia and asthma categories from the Coswara dataset were smaller and formed a balanced dataset of 48 asthma samples and 50 pneumonia samples.

The performance characteristics of the LDA and KNN classifiers are displayed in Table 3 using the area under the curve (AUC), accuracy, and Matthew’s correlation coefficient (MCC) metrics. While a receiver operating characteristic curve plots a true positive rate against a false positive rate, the AUC is a measure of a classifier’s overall performance. Likewise, accuracy is defined as the proportion of correctly classified samples. The MCC, however, ranges from $-1$ to $+1$, with a value of $+1$ representing a perfect prediction, 0 indicating a random prediction, and $-1$ indicating complete disagreement between the predicted and true labels. For COVID-19-positive versus COVID-19-negative LDA results, the best accuracy and AUC were achieved using the RP values of the respective frequency bands: 1100–1650 Hz and 300–800 Hz. The first frequency band, 1100–1650 Hz, corresponded to the optimal band identified previously, with the lowest Mann–Whitney U test *p* value. The second frequency band of interest, which was identified as an optimal band at 300–400 Hz, had the lowest accuracy and AUC of all the values, but they were still significant at 0.80 and 0.71, respectively. Similarly, the highest accuracy and AUC performance values for the classification of asthma versus pneumonia were achieved using the RP of the 50–900 Hz and 450–1000 Hz frequency bands, respectively. Again, the optimal frequency band identified in the Mann–Whitney U test differed from that for the highest LDA scores. However, the optimal band at 1400–1650 Hz exhibited sufficiently high levels of accuracy and AUC (0.89 and 0.90, respectively), indicating the ability of the LDA classifier to adequately distinguish between the pneumonia and asthma samples based on their respective RP values. For both the COVID-19-positive versus COVID-19-negative and pneumonia versus asthma samples, the LDA classifier did not reach optimal performance consistently for the previously identified optimal frequency bands based on the RP values. Nevertheless, it achieved adequate and comparable performance values for all the optimal bands, allowing us to deduce arguments similar to those for the KNN classifier performance values. Unlike the LDA classifier, for the COVID-19-positive group versus the control group, the 1100–1850 Hz band outperformed the optimal band within that region (i.e., 1100–1650 Hz), and the 300–400 Hz band had the highest overall performance, with an MCC value of 0.71. For the pneumonia versus asthma KNN classification, the low-frequency 50–900 Hz range yielded the highest MCC performance values, although both frequency bands in Region 1 had similar performance metric values. In short, the KNN classifier performed almost identically to the LDA classifier in the second region, with high metrics values for the 1400–1600 frequency band. Notably, this analysis was conducted using linear and non-linear machine-learning classifiers. This means that, altogether, these results corroborated our preliminary observations, indicating the existence of a set of desirable and optimal frequency bands in cough sounds that, using machine learning classifiers, we can leverage to detect and thus diagnose a cough’s underlying ailment.

**Table 3:** LDA and KNN classification performance results for the COVID-19-positive group versus the COVID-19-negative control group, and the pneumonia group versus the asthma group.

Region 1							Region 2						
Frequency band (Hz)	LDA			KNN			Frequency band (Hz)	LDA			KNN		
	Accuracy	AUC	MCC	Accuracy	AUC	MCC		Accuracy	AUC	MCC	Accuracy	AUC	MCC
[300, 400]	0.80	0.71	0.35	0.80	0.75	0.71	[1100, 1650]	0.88	0.75	0.77	0.75	0.62	0.58
[300, 600]	0.87	0.87	0.38	0.77	0.75	0.29	[975, 1750]	0.87	0.83	0.38	0.76	0.75	0.78
[300, 800]	0.85	0.90	0.60	0.77	0.67	0.47	[1100, 1850]	0.87	0.83	0.43	0.88	0.87	0.68

Region 1							Region 2						
Frequency band (Hz)	LDA			KNN			Frequency band (Hz)	LDA			KNN		
	Accuracy	AUC	MCC	Accuracy	AUC	MCC		Accuracy	AUC	MCC	Accuracy	AUC	MCC
[450, 1000]	0.89	0.93	0.82	0.89	0.87	0.65	[1400, 1600]	0.89	0.90	0.65	0.89	0.92	0.73
[50, 900]	0.92	0.86	0.81	0.88	0.83	0.82	[1450, 2100]	0.78	0.77	0.50	0.78	0.83	0.41

Performance metrics were accuracy, MCC, and AUC, and the classifier computations were based on the RP of signals within the frequency bands of interest. LDA refers to linear discriminant analysis, KNN refers to k-nearest neighbors, MCC refers to Matthew’s correlation coefficient and AUC refers to area under the ROC curve.

### Discussion

The application of the Mann–Whitney U test to our study data revealed statistically significant differences in the RP of cough sound signals between COVID-19-positive individuals and a COVID-19-negative control group, particularly in the 300–400 Hz and 1100–1650 Hz frequency bands. The notably low *p* values in these tests strongly suggest that these differences are not random occurrences, thereby supporting our hypothesis that the RP distributions in cough sound signals are distinctively different between the two groups. These results underscore the potential of RP in cough sound signals as a significant biomarker for COVID-19, especially within these specific frequency bands.

Further analysis using LDA for classification based on the RP of cough signals indicated a high level of accuracy in differentiating COVID-19-positive from COVID-19-negative patients across various frequency bands. Notably, the frequency band of 1100–1850 Hz demonstrated the highest classification accuracy, reaching a value of 1.88. Additionally, comparisons of RP values for pneumonia versus asthma across all relevant frequency bands showed substantial accuracy, exceeding 0.78. These findings reinforce the premise that the RP of cough signals is a reliable indicator for distinguishing not only between COVID-19 status but also between other respiratory conditions like pneumonia and asthma within these frequency ranges.

It is important to acknowledge that this study relied on crowdsourced data rather than clinical data. The cough recordings were self-reported by patients, which introduces a degree of uncertainty regarding their accuracy. Moreover, the control group was relatively small and lacked diversity, with a significant proportion of the dataset comprising male participants aged between 20 and 35 years, accounting for approximately 70% of the total sample. This demographic skew poses limitations on the study’s statistical significance and generalizability, indicating a need for more diverse and representative data in future research.

Additionally, the statistical methodologies employed in this study come with inherent limitations. For instance, the Welch method used for calculating the PSD provides only an approximation. These methodological constraints suggest that our findings should be considered preliminary, necessitating further research and validation.

Our study contributes novel insights into the field of respiratory disease diagnosis using cough sound signals, particularly for COVID-19, augmenting the existing body of literature that predominantly focuses on deep learning models like convolutional neural networks. Unlike previous studies which have reported varied model performance accuracy for COVID-19 prognosis, our approach employs classical machine learning classifiers and uniquely identifies specific frequency bands within the power density spectrum of cough signals for disease diagnosis. This methodological divergence not only confirms the potential of cough as a biomarker but also adds a new dimension to its analysis in the context of respiratory illnesses.

## Conclusion

This study highlights distinct physical and acoustical differences in cough sound signals linked to various respiratory diseases, with a focus on COVID-19. By comparing these signals with those from a control group of patients suffering from other respiratory ailments, we aimed to establish a benchmark for COVID-19 cough characteristics. While the limited size and diversity of the control group posed challenges, our findings significantly contribute to the understanding of cough sounds as biomarkers for respiratory conditions. We successfully identified specific low- and high-frequency bands associated with different diseases, demonstrating the potential of cough acoustics in diagnosing conditions like COVID-19, asthma, and pneumonia. This research represents an initial step towards a comprehensive understanding of cough sounds and their diagnostic potential.

## Data Availability

The dataset used in this study is publicly available and can be accessed and downloaded via the following link: https://github.com/iiscleap/Coswara-Data.
